# Involvement of protein kinase ζ in the maintenance of hippocampal long-term potentiation in rats with chronic visceral hypersensitivity

**DOI:** 10.1152/jn.00929.2014

**Published:** 2015-03-11

**Authors:** Aiqin Chen, Chengjia Bao, Ying Tang, Xiaoqing Luo, Lixia Guo, Bin Liu, Chun Lin

**Affiliations:** Fujian Medical University, School of Basic Medical Sciences, Laboratory of Pain Research, Key Laboratory of Brain Aging and Neurodegenerative Diseases, Neuroscience Research Center, Fuzhou City, Fujian Province, People's Republic of China

**Keywords:** chronic visceral pain, long-term potentiation, protein kinase M ζ, hippocampus, neonatal maternal separation

## Abstract

The hippocampal long-term potentiation (LTP) was implicated in the formation of visceral hypersensitivity in rats with irritable bowel syndrome in our previous study. Recent studies have shown that protein kinase M ζ (PKMζ) may be responsible for the maintenance of LTP in memory formation. However, it remains unclear whether PKMζ is involved in the visceral hypersensitivity. In this study, a rat model of visceral hypersensitivity was generated by neonatal maternal separation (NMS). The visceral hypersensitivity was assessed by recording responses of the external oblique abdominal muscle to colorectal distension. Our results demonstrated that hippocampal LTP and visceral hypersensitivity were enhanced significantly in rats of NMS. ζ-Pseudosubstrate inhibitory peptide (ZIP) could dose dependently inhibit the maintenance of Cornu Ammonis area 1 LTP in rats of NMS. Furthermore, Western blot data showed that the expression of hippocampal phosphorylated PKMζ (p-PKMζ) significantly increased in rats of NMS. In addition, bilateral intrahippocampal injections of ZIP attenuated the visceral hypersensitivity dose dependently in rats of NMS. The maximal inhibition was observed at 30 min, and significant inhibition lasted for 1.5–2 h after ZIP application. Besides, data from the open-field test and Morris water maze showed that ZIP did not influence the movement and spatial procedural memory in rats of NMS. In conclusion, p-PKMζ might be a critical protein in the maintenance of hippocampal LTP, which could result in visceral hypersensitivity.

irritable bowel syndrome (IBS) is a common, functional gastrointestinal disorder disease with the symptoms of repeated visceral pain and altered bowel habit (Canavan et al. 2014). Until now, the etiologies of recurrent visceral pain remain unclear. It is reported that early mental and physical injury is often linked with chronic functional visceral pain (Mayer et al. 2001; Rutten et al. 2014). It is popular knowledge that neonatal maternal separation (NMS) is considered a common model of psychological stress (Coutinho et al. 2002). However, the mechanism of chronic visceral pain induced by early psychical stress is still not completely understood.

It is acceptable that hippocampus, as an important area of the limbic system, is involved in several higher brain functions, including learning and memory (Bird and Burgess 2008; Jaffard and Meunier 1993). Long-term potentiation (LTP), as a model of synaptic plasticity changes, is believed to be the key to neuronal functions for learning and memory (Bliss and Collingridge 1993; Chen et al. 2012; Gerlai 2002). Chronic pain is a typical example of a synaptic plasticity change (Li et al. 2011; Price et al. 2006). Some of these permanent, central changes are the key reason for maintenance and recrudescence of chronic pain (Li et al. 2010; Luo et al. 2014). Chronic pain and memory are hypothesized to have tremendous similarities in the forming process and functional changes of the nervous system (Yi and Zhang 2011). Meanwhile, several studies support the views that the hippocampus plays a role in somatic pain (Liu and Chen 2009; Wei et al. 2000). However, whether hippocampal LTP is related to chronic visceral pain is still unknown.

Multiple protein molecules, such as phosphatidylinositol 3-kinases and PKC, have been confirmed to contribute to the induction of LTP (Ohnami et al. 2012; Yang et al. 2004). The protein kinase M ζ (PKMζ), an autonomously active, atypical PKC isoform, maintains persistent synaptic changes and memory storage (Li et al. 2010; Ling et al. 2002; Sacktor et al. 1993). In addition, PKMζ, in the hippocampus, plays an important role in sustaining spatial information and fear memory (Madronal et al. 2010; Sacktor 2012). Previous results from our laboratory revealed that hippocampal LTP induced by high-frequency stimulation (HFS) at Schaffer collateral (SC)-Cornu Ammonis area 1 (CA1) synapses significantly enhanced in IBS model rats (Chen et al. 2014). However, whether hippocampal PKMζ contributes to the maintenance of LTP in rats of NMS remains unsolved so far.

In this study, visceral hypersensitivity was evaluated by recording the amplitude of electromyography (EMG) to colorectal distension (CRD) in rats of NMS. The effects of ζ-pseudosubstrate inhibitory peptide (ZIP), a selective PKMζ inhibitor, on the inducement and maintenance of hippocampal LTP were tested by electrophysiological recordings in vitro. Furthermore, expressions of hippocampal PKMζ and its phosphorylation (p-PKMζ) were analyzed by the Western blotting method. In addition, the effects of bilateral intrahippocampal injections of ZIP on visceral hypersensitivity were evaluated with EMG measurements in vivo. Finally, the open-field test and water-maze experiments were used to examine whether ZIP had side effects on movement and learning memory.

## MATERIALS AND METHODS

### 

#### Animals.

Neonatal male Sprague-Dawley rats were purchased from the Department of Experimental Animal Center, Fujian Medical University (Fujian Province, China). The neonatal rats were with their mother until they were 21 days old. The weaned rats were bred for 8 wk before the experiments. The rats were housed six/cage, in large cages before surgery and individually in small cages after surgery. All of the programs of animal experiments followed the guidelines of the International Association for the Study of Pain (Zimmermann 1983) and were conducted in accordance with the Animal Care and Use Committee of and approved by Fujian Medical University.

#### NMS procedure.

The NMS procedure was adapted from a protocol described previously (Coutinho et al. 2002). In brief, the litters that were randomly assigned to undergo NMS were taken away from the home cage and moved to a separate, clean cage for 3 h. The cages were situated on a heating pad (30–33°C) to keep the pups at a temperature similar to the mother's external body temperature. Later, the pups were returned to their mothers. This procedure was repeated for *postnatal days 3–21*. The controls remained undisturbed in the home cage with their mothers.

#### Assessment of visceral hypersensitivity.

Visceral sensitivity was evaluated by EMG to graded CRD (20, 40, 60, and 80 mmHg), as described in many previous studies (Chen et al. 2014; Kannampalli et al. 2014; Luo et al. 2014; Saab et al. 2006). In brief, the animals were lightly anesthetized with isoflurane by a Matrx VMR anesthesia machine (Midmark, Dayton, OH). The balloon was inserted into the colon and fixed to the rat's tail. Two silver, bipolar electrodes were placed in the external oblique muscle of the abdomen. The balloon was inflated to each CRD for 10 s, followed by 4 min of rest. The magnitude of EMG activity was measured by a RM6240BD multichannel physiological signal acquisition and processing system (Chengdu Instrument Factory, ChengDu, China). The EMG was recorded three times for each CRD. The average of the three recordings was used as the magnitude of EMG activity for each CRD. The EMG data of 10 s before CRD (baseline) and 10 s during CRD (response) were recorded for calculation. The responses were normalized as the percentage of increased EMG amplitude during CRD over the baseline, i.e., (response − baseline)/baseline × 100%.

#### Surgery and bilateral intrahippocampus injections.

The experimental procedures were performed as described previously (Chen et al. 2014). In brief, the hippocampal CA1 region was located by stereotaxic instrument (Narishige, Tokyo, Japan), according to the methods published by Paxinos and Watson (1998): 4.0 mm posterior to the bregma, 2.8 mm lateral to the midline, and 2.5 mm beneath the surface of the skull. After a 7-day recovery period from surgery, two stainless-steel syringes (0.4 mm in diameter) were inserted through a guide cannula to produce 0.5 mm below the tips of the latter. Then, aliquots of 2 μl vehicle or ZIP (Tocris Bioscience, Bristol, UK) were injected into both sides of the CA1 region within 5 min, according to our protocol. The needles were left in place for an additional 5 min to allow for diffusion of the injected solution.

#### Hippocampal slice preparation and electrophysiological recordings.

Hippocampal slices were prepared according to the methods described previously (Chen et al. 2012). Slices were incubated on a mesh net submerged in oxygenated artificial cerebrospinal fluid and recovered for at least 1 h. The field excitatory postsynaptic potentials (fEPSPs) were recorded from the dendritic layer of the CA1 pyramidal cells by stimulating the SC using RM6240BD. The recording electrodes were filled with NaCl (3 M). The strength of synaptic transmission was estimated by calculating the initial amplitude and slope of fEPSPs. The strength of stimulation was adjusted to 50% of the maximum fEPSP-amplitude values. Baseline values were recorded at least 10 min. LTP was induced by two trains of HFS (100 pulses at 100 Hz with an intertrain interval of 10 s).

#### Open-field test.

The open-field test assessed the effects of bilateral intrahippocampal injections of ZIP on the movement in rats of NMS. The dimensions of the box were 100 × 100 × 60 cm. Each rat was lightly settled in the center area of the open field, and its movement locus was videotaped for 5 min. The rats were tested at 30 min after intrahippocampal administration of 5.0 nM ZIP. The measurements included the total walking distance (meters) and the average speed (centimeters/second). After each test, the box was swept entirely to erase the effects of odor.

#### Morris water maze.

The pool was 160 cm in diameter and 60 cm in height and was filled with water at 25°C to avoid hypothermia. The pool was divided into quadrants. The escape platform was hidden, 2 cm beneath the water surface, and placed in the middle of the southeast quadrant throughout the training session. Some landmarks were fixed to the walls of the water-maze room to help rats find the platform. The rats finding the escape platform within 60 s were left on it for 20 s. However, the rats that were unsuccessful in finding the platform in a latency time of 60 s were artificially placed on the platform for 20 s. The rats were trained for 5 days with one, four-trial block/day. On *day 6*, the platform was removed to test spatial accuracy and spatial procedural memory. The performance of the rats was monitored with an overhead video camera connected to an image analyzer and analyzed by the water-maze RD 1101-MWM software (Shanghai Mobiledatum, Shanghai, China). The experiments were performed at 10 AM. The rats were tested at 30 min after intrahippocampal administration of 5.0 nM ZIP.

#### Western blotting.

Equal quantities of each sample (30 μg) from the hippocampus were submitted to the gel for separating. Then the proteins were transferred to polyvinylidene difluoride membranes (Invitrogen, Carlsbad, CA). Membranes were incubated with the following primary antibodies: rabbit anti-PKMζ MAb (1:500; Santa Cruz Biotechnology, Santa Cruz, CA), rabbit anti-p-PKMζ MAb (1:400; Santa Cruz Biotechnology)(King et al. 2012; Marchand et al. 2011), and rabbit anti-GAPDH primary antibody (1:3,000; Bioworld Technology, St. Louis Park, MN). The membranes were then incubated with peroxidase-conjugated anti-rabbit IgG (1:1,000; Abcam, Cambridge, MA).

#### Statistical analysis.

All data were expressed as the means ± SE. The two independent samples *t*-test was used to determine whether there was a significant difference in the response to CRD pressure between NMS and control rats. CRD data, after bilateral intrahippocampal injections of compounds, were analyzed by one-way repeated-measures ANOVA, followed by Bonferroni post hoc test. CRD data, before and after injection, were compared and analyzed using the paired *t*-test. Western blot results were analyzed by the two independent samples *t*-test. LTP was measured 60 min after HFS and reported as the mean ± SE of baseline fEPSP amplitude or slope. The two independent samples *t*-test was used to compare the hippocampal field potential of NMS and control rats. One-way ANOVA, followed by Dunnett's post hoc test, was used to compare the effects of ZIP on LTP in rats. The two independent samples *t*-test was used to observe the effects of ZIP on spontaneous locomotor activity and learning memory in rats of NMS. *P* < 0.05 was considered statistically significant.

## RESULTS

### 

#### NMS results in visceral hypersensitivity and facilitation of hippocampal LTP in rats.

**Fig. 1.**
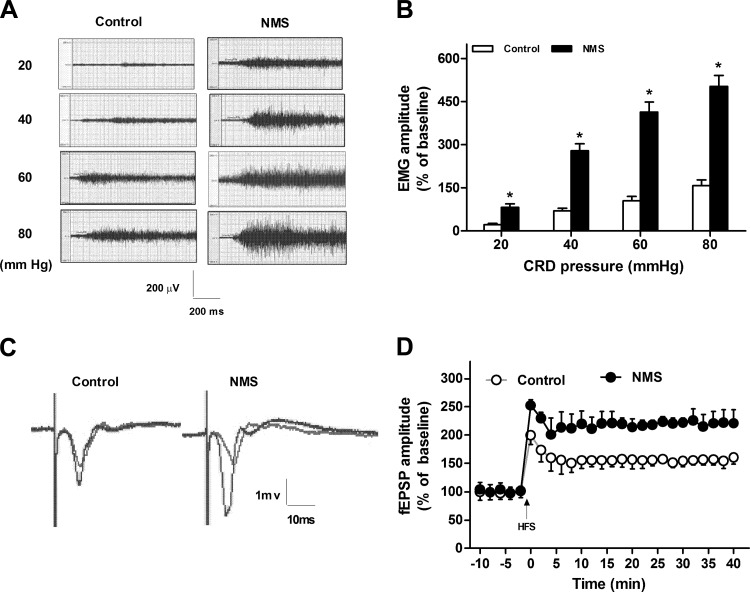
Visceral hypersensitivity and enhanced hippocampal long-term potentiation (LTP) in rats of neonatal maternal separation (NMS). *A*: the original, typical recording of electromyography (EMG) in rats under 20–80 mmHg colorectal distension (CRD) pressure. *B*: the statistical graph of the percentage of electromyographic amplitude over baseline under 20–80 mmHg CRD in rats; *n* = 8. *P < 0.05 vs. controls. *C*: the original, typical recording of field excitatory postsynaptic potentials (fEPSPs) in rats. *D*: the standardized fEPSP amplitude in rats. LTP was induced by 2 trains of high-frequency stimulation (HFS; 100 pulses at 100 Hz, with an intertrain interval of 10 s).

Visceral hypersensitivity was assessed by recording the response of EMG in rats at 8 wk of age. The original, typical recording of EMG in rats under 20–80 mmHg CRD pressure is showed in Fig. 1*A*. The magnitude of EMG to graded CRD pressure was significantly higher in rats of NMS than that in control rats (two independent samples *t*-test, *P* < 0.05; Fig. 1*B*). Visceral sensitivity in rats of NMS significantly increased by 272% under 20 mmHg CRD, 296% under 40 mmHg CRD, 297% under 60 mmHg CRD, and 219% under 80 mmHg CRD. These results suggest that neonatal NMS significantly increased the visceromotor response to CRD in adult rats when compared with that in controls.

Examples of fEPSP traces were obtained at 10 min pre-HFS and 40 min post-HFS (Fig. 1*C*). The strength of stimulation was adjusted to 50% of the maximum fEPSP-amplitude values. LTP was induced by two trainings of HFS (100 pulses at 100 Hz, with an intertraining interval of 10 s). This would trend the LTP toward a nonprotein, synthesis-dependent form. The fEPSP slope and fEPSP amplitude (Fig. 1*D*) were enhanced by 144% and 89%, respectively, in rats of NMS when compared with that in the controls (two independent samples *t*-test, *P* < 0.05). These results suggested that NMS resulted in the facilitation of the CA1 hippocampal LTP.

#### Inhibitory effects of ZIP on the maintenance of CA1 hippocampal LTP in rats of NMS in vitro.

**Fig. 2.**
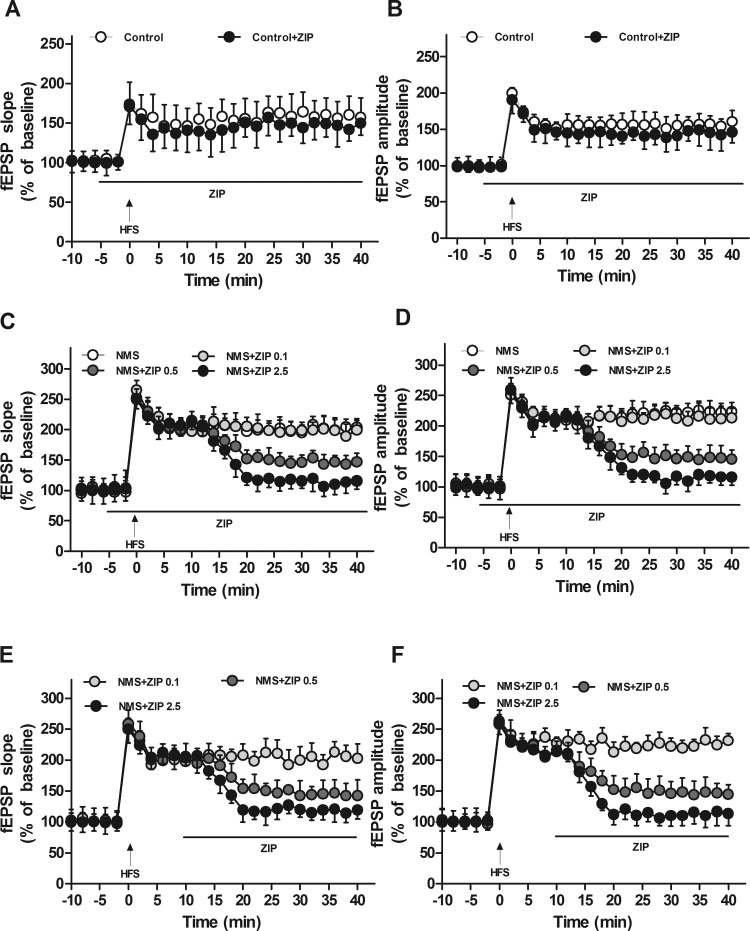
Effects of ζ-pseudosubstrate inhibitory peptide (ZIP) on field-potential LTP of hippocampus slices in rats. *A*: the effect of ZIP on the standardized fEPSP slope in control rats. *B*: the effect of ZIP on the standardized fEPSP amplitude in control rats. *C*: the effect of ZIP at 10 min pre-HFS on the standardized fEPSP slope in rats of NMS. *D*: the effect of ZIP at 10 min pre-HFS on the standardized fEPSP amplitude in rats of NMS. *E*: the effect of ZIP at 10 min post-HFS on the standardized fEPSP slope in rats of NMS. *F*: the effect of ZIP at 10 min post-HFS on the standardized fEPSP amplitude in rats of NMS. LTP was induced by 2 trains of HFS (100 pulses at 100 Hz, with an intertrain interval of 10 s); *n* = 8 slices from 4 rats for each group.

To examine whether PKMζ was required for the CA1 hippocampal LTP of chronic visceral pain, the effect of ZIP, a selective PKMζ inhibitor, on hippocampal LTP was examined in rats. Bath application of ZIP into the brain slices at 10 min pre-HFS had no significant influence on the inducement and maintenance of CA1 LTP in controls (ANOVA/Dunnett's, *P* > 0.05; Fig. 2, *A* and *B*). ZIP (0.1, 0.5, and 2.5 μM) had no significant effects on basal synaptic responses and fEPSP during 15 min post-HFS in rats of NMS (*P* > 0.05; Fig. 2, *C* and *D*). However, 0.5 μM ZIP reduced the fEPSP amplitude and slope by 61% (from 122.44 ± 12.99% to 46.84 ± 12.96%) and 62% (from 118.74 ± 10.79% to 44.64 ± 14.09%), respectively, at 40 min post-HFS (*P* < 0.05; Fig. 2, *C* and *D*). Similarly, 2.5 μM ZIP inhibited the amplitude and slope of fEPSPs by 89% (from 122.44 ± 12.99% to 13.43 ± 10.27%) and 87% (from 118.74 ± 10.79% to 14.27 ± 13.38%), respectively, at 40 min post-HFS (*P* < 0.05; Fig. 2, *C* and *D*). However, 0.1 μM ZIP had no significant influence on fEPSPs in rats of NMS (*P* > 0.05; Fig. 2, *C* and *D*).When compared with the 0.1 μM ZIP, 0.5 μM ZIP decreased fEPSP amplitude and slope by 58% and 53%, and when compared with the 0.5 μM ZIP, 2.5 μM ZIP decreased fEPSP amplitude and slope by 71% and 68%, respectively. The effects of ZIP on the CA1 hippocampal LTP presented a dose-dependent suppression (*P* < 0.05; Fig. 2, *C* and *D*). The application of ZIP at 10 min post-HFS had the same effects on LTP as at 10 min pre-HFS (Fig. 2, *C–F*). These results suggest that PKMζ plays an essential role in the maintenance of LTP in the hippocampal CA1 region of rats of NMS.

#### The upregulation of hippocampal p-PKMζ protein in rats of NMS.

**Fig. 3.**
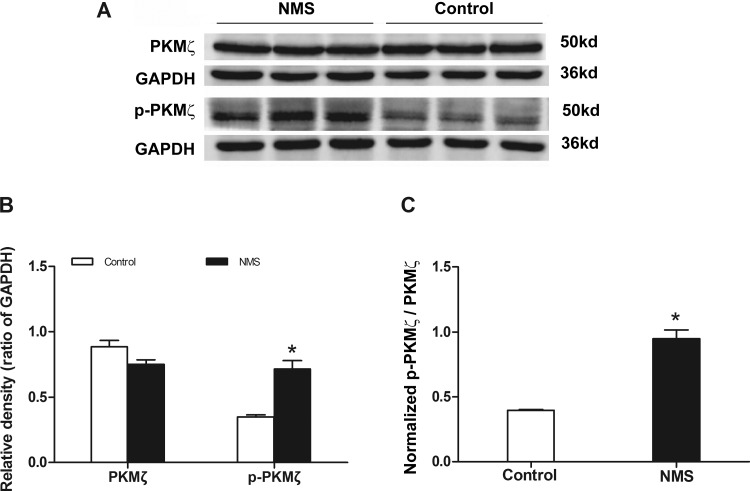
Expression of hippocampal protein kinase M ζ (PKMζ) and phosphorylated PKMζ (p-PKMζ) in rats. *A*: Western blot results displayed the protein level of hippocampal PKMζ and p-PKMζ in rats. *B*: statistical graph displayed that level of PKMζ and p-PKMζ in rats. The expression of p-PKMζ increased significantly in the rats of NMS, *n* = 3. *P < 0.05 vs. controls. *C*: the ratio of normalized p-PKMζ over PKMζ in hippocampus was increased significantly in rats of NMS. *P < 0.05 vs. controls.

PKMζ is activated by phosphorylation. The expression and phosphorylation of PKMζ in the hippocampus were determined with the Western blotting technique. The results showed that the level of p-PKMζ protein markedly increased by 50% (from 0.35 ± 0.01 to 0.71 ± 0.06) in rats of NMS (two independent samples *t*-test, *P* < 0.05; Fig. 3*B*). There was no significant difference in the expression of hippocampal PKMζ protein between rats of NMS (0.75 ± 0.05) and the controls (0.89 ± 0.05, *P* > 0.05; Fig. 3*B*). The ratio of normalized p-PKMζ over PKMζ in the hippocampus was increased significantly by 138% (from 0.40 ± 0.01 to 0.95 ± 0.07) in rats of NMS (*P* < 0.05; Fig. 3*C*).

#### Bilateral intrahippocampal injections of ZIP attenuated visceral hypersensitivity in rats of NMS.

**Fig. 4.**
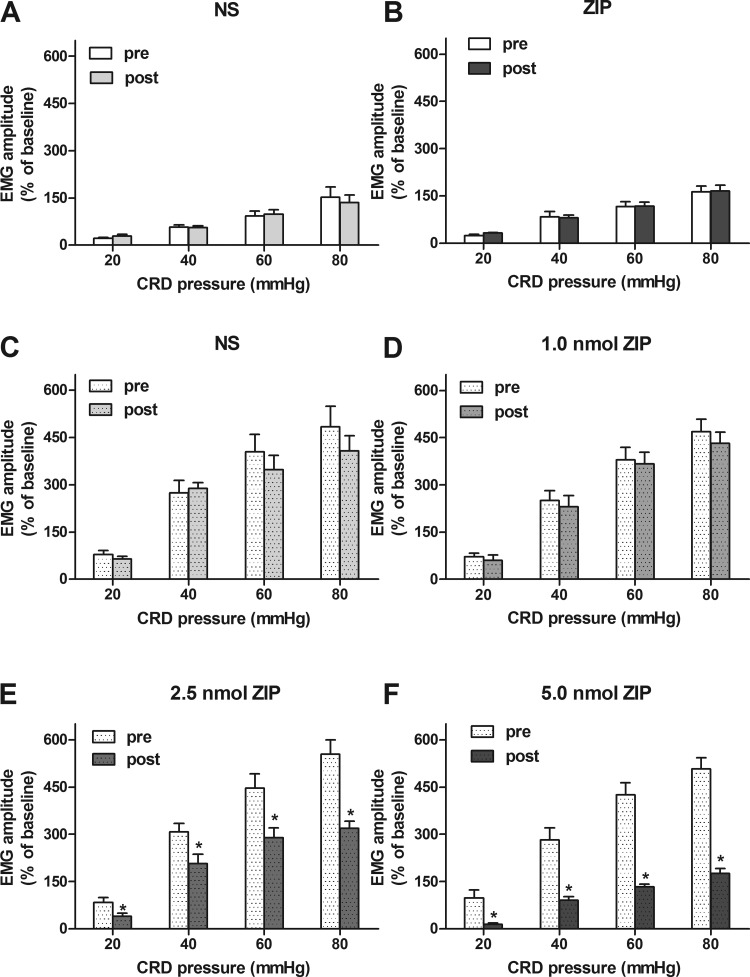
Effect of bilateral intrahippocampal injections of ZIP on EMG in rats. *A*: the statistical graph of the electromyographic amplitude after bilateral intrahippocampal injections of normal saline (NS; 2 μl each side) in controls. *B*: the statistical graph of the electromyographic amplitude after bilateral intrahippocampal injections of 5.0 nM ZIP (2 μl each side) in controls. Bilateral intrahippocampal injections of ZIP had no influence on EMG in controls. *C*: the statistical graph of the electromyographic amplitude after bilateral intrahippocampal injections of NS (2 μl each side) in rats of NMS. *D–F*: the statistical graph of the electromyographic amplitude after bilateral intrahippocampal injections, 1, 2.5, and 5 nM ZIP (2 μl each side) in rats of NMS. ZIP dose dependently inhibited the visceral hypersensitivity in rats of NMS; *n* = 8. *P < 0.05 vs. pre-drug.

Bilateral intrahippocampal injections of ZIP (5 nM, 2 μl each side) had no effect on the responses of EMG to 20–80 mmHg CRD in controls (paired *t*-test, *P* > 0.05; Fig. 4, *A* and *B*). There was no obvious change in responses of EMG to 20–80 mmHg CRD after an injection of 1 nM ZIP when compared with before in rats of NMS (paired *t*-test, *P* > 0.05; Fig. 4*D*). The responses of EMG to 20, 40, 60, and 80 mmHg CRD were decreased by 52, 33, 35, and 42% after an injection of 2.5 nM ZIP compared with before in rats of NMS (*P* < 0.05; Fig. 4*E*). The EMG responses to 20, 40, 60, and 80 mmHg CRD were decreased by 86, 68, 68, and 65%, respectively, after injection of 5.0 nM ZIP compared with the responses of NMS rats before injection (*P* < 0.05; Fig. 4*F*).

The inhibitory effects of 2.5 nM ZIP were enhanced by 238, 315, 997, and 450% under 20, 40, 60, and 80 mmHg when compared with the effects of 1.0 nM ZIP in NMS rats (ANOVA/Bonferroni *t*-test, *P* < 0.05; Fig. 4, *D* and *E*). The inhibitory effects of 5.0 nM ZIP were enhanced by 458, 753, 2,028, and 748% under 20, 40, 60, and 80 mmHg when compared with 1.0 nM ZIP in NMS rats (*P* < 0.05; Fig. 4, *D* and *F*). The effects of ZIP on visceral hypersensitivity presented a dose-dependent suppression.

#### Time profiles of the inhibitory effect of ZIP on visceral hypersensitivity in NMS rats.

**Fig. 5.**
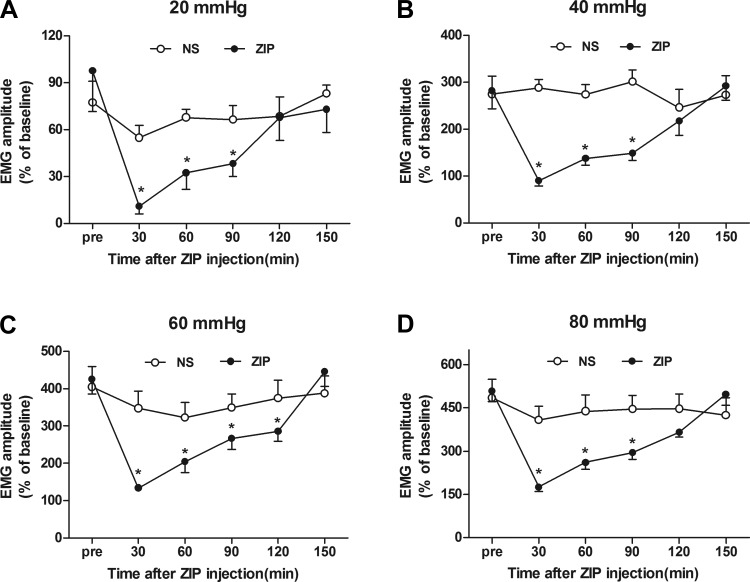
Duration curve of suppressive action of 5 nM ZIP on visceral sensitivity in rats of NMS. *A–D*: the duration curve of the action of 5 nM ZIP on EMG under 20, 40, 60, and 80 mmHg CRD in rats of NMS. The maximal suppression was recorded at 30 min after ZIP administration in rats of NMS; *n* = 8. *P < 0.05 vs. pre-drug.

To observe further the time profiles of ZIP, EMG responses to 20–80 mmHg CRD were recorded every 30 min in a 150-min period after intrahippocampal injection of 5.0 nM ZIP in NMS rats. The responsive electromyographic amplitudes to 20 mmHg CRD were inhibited by 88, 66, 60, and 0%, respectively, at 30, 60, 90, and 120 min (Fig. 5*A*). The responsive electromyographic amplitudes to 40 mmHg CRD were inhibited by 68, 51, 47, and 22% (Fig. 5*B*). The responsive electromyographic amplitudes to 60 mmHg CRD were inhibited by 68, 51, 37, and 32% (Fig. 5*C*). The responsive electromyographic amplitudes to 80 mmHg CRD were inhibited by 65, 48, 41, and 28% (Fig. 5*D*). These findings indicated that the maximal suppression efficiency of ZIP was recorded at 30 min and that significant inhibition lasted for ∼120 min after the injection of ZIP.

#### Effects of bilateral intrahippocampal injections of ZIP on spontaneous locomotor activity and learning memory in rats.

The open-field test was dedicated to determining the effect of bilateral intrahippocampal injections of ZIP on spontaneous locomotor activity in rats. ZIP had no effect on the walking distance [Control + normal saline (NS): 17.37 ± 4.30 m vs. Control + ZIP: 17.00 ± 5.07 m; NMS + NS: 14.72 ± 5.22 m vs. NMS + ZIP: 14.60 ± 4.09 m; two independent samples *t*-test, *P* > 0.05] and average speed (Control + NS: 5.46 ± 1.42 cm/s vs. Control + ZIP: 6.94 ± 2.14 cm/s; NMS + NS: 5.54 ± 1.91 cm/s vs. NMS + ZIP: 4.87 ± 1.36 cm/s, *P* > 0.05) compared with the NS group, indicating that bilateral intrahippocampal injections of ZIP had no significant influence on spontaneous motor activity in rats.

**Fig. 6.**
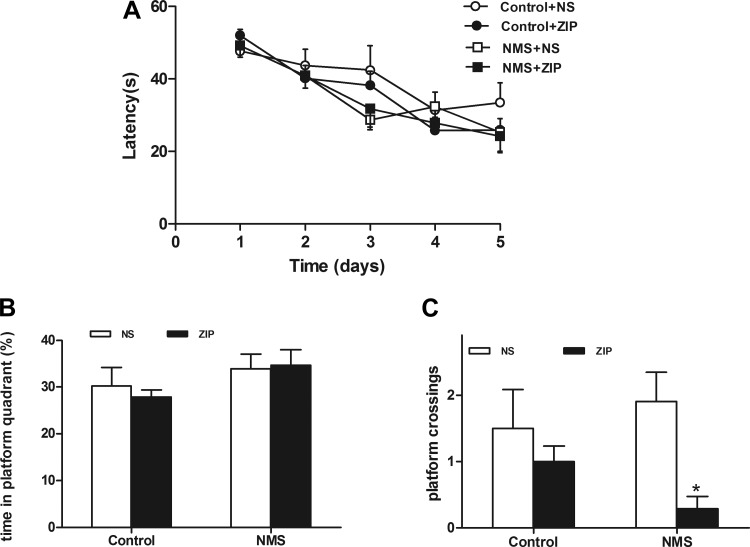
Effects of intrahippocampal administration of ZIP on spatial procedural memory and spatial accuracy in rats. *A*: escape latencies during training for 5 days in rats. *B*: effects of ZIP on time spent in quadrant with platform in rats. Bilateral intrahippocampal injections of ZIP had no influence on spatial procedural memory in rats. *P* > 0.05 Control + ZIP vs. Control + NS; NMS + ZIP vs. NMS + NS. *C*: effects of ZIP on the numbers of platform crossings in rats. ZIP could decrease the numbers of platform crossings in rats of NMS but not in controls. *P < 0.05 NMS + ZIP vs. NMS + NS; *P* > 0.05 Control + ZIP vs. Control + NS.

The Morris water maze was used to test the effect of bilateral intrahippocampal injections of ZIP on spatial accuracy and spatial procedural memory in rats. There were no differences in rats' escape latencies during the 5-day training (Fig. 6*A*). The application of NS and ZIP in rats did not result in significant differences in the time the rats spent in the platform quadrant (two independent samples *t*-test, *P* > 0.05; Fig. 6*B*). The results in rats of NMS showed that ZIP had an influence on spatial accuracy (*P* < 0.05; Fig. 6*C*). However, ZIP did not affect the spatial accuracy in controls (*P* >0.05; Fig. 6*C*).

## DISCUSSION

In the previous studies, HFS-induced LTP at SC-CA1 synapses significantly increased in the rats of IBS (Chen et al. 2014). In the present study, we demonstrate further that PKMζ plays an essential role in the maintenance of hippocampal LTP and visceral hypersensitivity in rats of NMS. Firstly, the visceral hypersensitivity and hippocampal LTP were significantly enhanced in rats of NMS. Secondly, the application of ZIP could dose dependently block the maintenance of LTP in the hippocampal CA1 region in rats of NMS. Thirdly, expressions of the hippocampal p-PKMζ protein significantly increased in rats of NMS. Finally, the injection of ZIP significantly attenuated visceral hypersensitivity without obvious side effects on the movement and spatial procedural memory in rats of NMS. However, ZIP seems to decrease the spatial accuracy, although not in the controls. These results revealed that PKMζ was crucial for the maintenance of long-term plasticity in the hippocampus and could contribute to visceral hypersensitivity induced by NMS.

### 

#### NMS facilitates hippocampal LTP and chronic visceral hypersensitivity.

In this study, model rats were established by NMS for 3 h daily during *postnatal days 3–21*, and the visceral hypersensitivity significantly enhanced when they were adults. Our results, together with the references, supported the viewpoint that early life stress may trigger visceral hypersensitivity (Chen et al. 2014; Hu et al. 2013; Ren et al. 2007; Yang et al. 2012). In addition, there is sufficient clinical evidence to indicate a close relationship between psychosocial factors and visceral hypersensitivity (Mayer et al. 2001; Rutten et al. 2014). Ren et al. (2007) have also addressed that NMS, as a risk factor, results in permanent changes in the central nervous system in the rats of IBS. In our study, the expression of hippocampal p-PKMζ significantly increased, and ZIP could significantly inhibit visceral hypersensitivity and hippocampal LTP in rats of NMS. It is postulated that NMS could contribute to permanent alterations of synaptic plasticity, which results in visceral hypersensitivity.

In our study, the increase of LTP is ∼50% in controls, which is close to results in Song et al. (2012). However, the LTP observed after NMS is >100% the baseline (Fig. 1*D*), which is similar to several recent studies (Chen et al. 2014; Serrano et al. 2005; Sousa et al. 2014). We conjecture that NMS may contribute to some permanent alterations of synaptic plasticity, resulting in the increase of LTP. The magnitude of hippocampal LTP was significantly higher in 8-wk-old rats of NMS than that in the age-matched controls in this study. Our results are coincidental with the reports of somatic pain that peripheral-persistent nociception amplified the magnitude of LTP in the hippocampus (Liu et al. 2012; Lyu et al. 2013). These findings support the view that there could be some similar hippocampal mechanisms between somatic and visceral pain. However, these changes conflicted with the report on the hippocampal plasticity in NMS rats, where Sousa et al. (2014) reported that NMS rats had an associated lower magnitude of hippocampal LTP when rats reached 70 wk of age. The main factor for the conflicts could be the obvious differences in the ages of the rats (70 vs. 8 wk). There are minor differences in stimulation parameters and the period of NMS, which could contribute less to the conflicts.

#### PKMζ contributes to hippocampal LTP and visceral hypersensitivity.

A remarkable report was that hippocampal PKMζ was required for the maintenance of spatial information and fear memory (Madronal et al. 2010; Pastalkova et al. 2006). It is reported that a peripheral nerve injury triggered activation of PKMζ in the anterior cingulate cortex, and a local microinjection of ZIP erased synaptic potentiation and inhibited behavioral sensitization (Li et al. 2010). In the present study, the level of hippocampal p-PKMζ significantly increased in rats of NMS. Meanwhile, ZIP significantly attenuated visceral hypersensitivity in rats of NMS, suggesting that hippocampal p-PKMζ could take part in the formation of visceral hypersensitivity. However, our results are inconsistent with the report that the expression of hippocampal PKMζ and p-PKMζ did not vary in neuropathic pain (Li et al. 2010). These differences indicate that the effects of hippocampal PKMζ vary in different types of pain. ZIP significantly attenuated visceral hypersensitivity for ∼2 h in rats of NMS in our study. The return of sensitivity may be due to ongoing input from regions outside of the hippocampus, which reverses the effects of ZIP and may result in the EMG returning to the baseline in a few hours. The attenuated effect of ZIP to visceral pain was another possible reason. In addition, the analgesic effects of ZIP lasted for at least 2 h, but there was a recovery at 24 h after the injection (Li et al. 2010). Some studies reported that ZIP could persistently erase memory (Pastalkova et al. 2006; Serrano et al. 2008). It is implied that there might be some differences between the effects of PKMζ on pain and memory. These findings indicate that the hippocampus may be one of the integral components in the modulation of pain in accord with several previous studies (Chen et al. 2014; Liu et al. 2011, 2012).

Serrano et al. (2005) studied the mechanism of LTP and found that PKMζ was one of the key factors in maintaining LTP. However, the roles of PKMζ in LTP and memory might be controversial (Volk et al. 2013). Hippocampal LTP induced the product of PKMζ, and p-PKMζ is an important player in LTP and memory storage (Pastalkova et al. 2006; Shema et al. 2009). More interestingly, both hippocampal LTP and p-PKMζ were increased in rats of NMS, whereas the level of total PKMζ remained unchanged. The results indicate that the increase of LTP induced by NMS may be a novel activation of pre-existing PKMζ by increased phosphorylation, rather than new synthesis. Certainly, further investigation is needed to distinguish the above mechanisms. In addition, ZIP had little or no effect on the LTP of hippocampal slices in controls, which is in accordance with the report that ZIP did not affect an early phase of LTP (Serrano et al. 2005). These results indicate the early LTP is not mediated by PKMζ and not sensitive to ZIP at the concentrations used in controls (Ren et al. 2013; Serrano et al. 2005). Certainly, the activation of PKCι/λ, which mediates a form of early LTP, might have occurred as well (Ren et al. 2013). However, ZIP could dose dependently inhibit the maintenance of hippocampal LTP in rats of NMS. Thus the involvement of PKMζ in the maintenance of LTP could also be an underlying mechanism of chronic pain in agreement with memory (Li et al. 2010; Ohnami et al. 2012).

In addition, bilateral intrahippocampal injections of ZIP did not work significantly on the movement and spatial procedural memory in rats of NMS. The results support the viewpoint that the inhibitive effect of ZIP on pain may be selective (Li et al. 2010). The report showed effects of ZIP on spatial accuracy (decreased number of platform crossings) and not on spatial procedural memory (time in the platform quadrant) (Serrano et al. 2008). There are some differences between our results and the report. Our results indicated that ZIP did not affect the number of platform crossings and time in the platform quadrant in the controls. The reason might be that the dosages of ZIP in our study are much less than that in the report. Interestingly, the same dosage of ZIP could decrease the number of platform crossings but not affect the time in the platform quadrant in rats of NMS. Therefore, we postulated that the increase of hippocampal p-PKMζ and LTP in rats of NMS may lead to the above results, but the plausible mechanism needs further exploration.

#### Correlations between hippocampal LTP and pain.

There is a popularly acceptable idea that hippocampal LTP, as a cellular model for synaptic plasticity, contributes to learning memory (Bliss and Collingridge 1993; Li et al. 2010). It is proposed that synaptic plasticity is a key mechanism for chronic somatic pain (Li et al. 2010). It is reported that pain and memory may have a surprising likeness in synaptic plasticity (Ji et al. 2003). In addition, it is acceptable that chronic pain is a nociceptive memory (Yi and Zhang 2011). A large number of clinical studies have shown that harmful pain and its emotion may give rise to the chronic pain memory lasting for many years after injury (Choi et al. 2007; Noel et al. 2012; Sanchez 2011).

An interesting and probably important observation in this study was a significant enhancement in hippocampal LTP in rats of NMS. The enhancement may be largely attributed to the susceptibility of the hippocampus to ongoing neuronal and circuit maturation in the early postnatal stages (Sousa et al. 2014). Our results implied that rats of NMS might facilitate chronic visceral pain-related memory when they age. It reinforces a view that there could be similar mechanisms between hippocampal LTP contributing to pain and memory (Ji et al. 2003). The enhanced magnitude of LTP was demonstrated in the hippocampal slices of rats with chronic somatic pain (Liu et al. 2012; Lyu et al. 2013). Besides, central sensitization and LTP in the hippocampus share some resemblance to molecular mechanisms (Ji et al. 2003). These reports support the view that there could be some correlations between hippocampal LTP and chronic pain. In addition, in rats of NMS, ZIP could significantly inhibit hippocampal LTP in vitro and visceral hypersensitivity in vivo. Altogether, these results implied that hippocampal LTP may be one of the key factors for a persistence of chronic visceral pain. We conjecture that chronic pain memory may facilitate the responses to the stimulation and result in hypersensitivity or allodynia. Further studies to investigate the relationships between the hippocampus roles in chronic visceral pain in hypersensitivity and in memory of chronic pain experiences may be warranted.

## GRANTS

Support for the present work was provided by grants from the National Natural Science Foundation of China (81471138 and 81100998) and Natural Science Foundation of Fujian Province (2014J01124).

## DISCLOSURES

The authors declare that they have no competing interests.

## AUTHOR CONTRIBUTIONS

Author contributions: C.L. conception and design of research; A.C., C.B., Y.T., X.L., L.G., and B.L. performed experiments; A.C., Y.T., X.L., L.G., and C.L. analyzed data; B.L. and C.L. interpreted results of experiments; A.C. and C.B. prepared figures; C.B. drafted manuscript; C.L. edited and revised manuscript; C.L. approved final version of manuscript.
